# Characterization and Expression Analysis of Phytoene Synthase from Bread Wheat (*Triticum aestivum* L.)

**DOI:** 10.1371/journal.pone.0162443

**Published:** 2016-10-03

**Authors:** Flowerika, Anshu Alok, Jitesh Kumar, Neha Thakur, Ashutosh Pandey, Ajay Kumar Pandey, Santosh Kumar Upadhyay, Siddharth Tiwari

**Affiliations:** 1 National Agri-Food Biotechnology Institute (NABI), Department of Biotechnology, Ministry of Science and Technology (Government of India), C-127, Industrial Area, Phase VIII, S.A.S. Nagar, Mohali, 160071, Punjab, India; 2 Department of Botany, Panjab University, Chandigarh, India-160014; 3 Department of Biotechnology, Panjab University, Chandigarh, India-160014; Huazhong University of Science and Technology, CHINA

## Abstract

Phytoene synthase (PSY) regulates the first committed step of the carotenoid biosynthetic pathway in plants. The present work reports identification and characterization of the three *PSY* genes (*TaPSY1*, *TaPSY2* and *TaPSY3*) in wheat (*Triticum aestivum* L.). The *TaPSY1*, *TaPSY2*, and *TaPSY3* genes consisted of three homoeologs on the long arm of group 7 chromosome (7L), short arm of group 5 chromosome (5S), and long arm of group 5 chromosome (5L), respectively in each subgenomes (A, B, and D) with a similarity range from 89% to 97%. The protein sequence analysis demonstrated that TaPSY1 and TaPSY3 retain most of conserved motifs for enzyme activity. Phylogenetic analysis of all TaPSY revealed an evolutionary relationship among PSY proteins of various monocot species. TaPSY derived from A and D subgenomes shared proximity to the PSY of *Triticum urartu* and *Aegilops tauschii*, respectively. The differential expression of *TaPSY1*, *TaPSY2*, and *TaPSY3* in the various tissues, seed development stages, and stress treatments suggested their role in plant development, and stress condition. *TaPSY3* showed higher expression in all tissues, followed by *TaPSY1*. The presence of multiple stress responsive *cis*-regulatory elements in promoter region of *TaPSY3* correlated with the higher expression during drought and heat stresses has suggested their role in these conditions. The expression pattern of *TaPSY3* was correlated with the accumulation of β-carotene in the seed developmental stages. Bacterial complementation assay has validated the functional activity of each TaPSY protein. Hence, *TaPSY* can be explored in developing genetically improved wheat crop.

## Introduction

Carotenoids are the natural pigments that constitute important components in all photosynthetic organisms. They are involved in harvesting light energy and provide protection to photosynthetic apparatus against reactive oxygen species [1]. Carotenoids also act as precursors of signalling molecules that influence development and respond to the biotic/abiotic stresses [2]. Carotenoids confer various health benefits to humans by preventing macular degeneration, certain type of cancers and various age related disorders [3–4]. In carotenoids, the β-carotene is a major precursor for vitamin A biosynthesis along with α-carotene and β-cryptoxanthin [5]. In plants, carotenoids provide specific colour to flowers and fruits to attract insects and animals [6]. They are synthesized within the plastids by nuclear encoded enzymes [6–7]. Carotenoid biosynthesis commences with the formation of phytoene from geranylgeranyl pyrophosphate (GGPP), a first step in the pathway which is catalysed by phytoene synthase (PSY) [8–9]. Four double bonds are introduced into phytoene by phytoene desaturase (PDS) and ζ-carotene desaturase (ZDS), through two symmetric dehydrogenation steps to yield ζ-carotene and lycopene, respectively [1]. The PSY is known to control carotenoid flux in seeds and catalyzes the first committed step of carotenogenesis [6,7,10]. Gene duplication events in *PSY* are prevalent in grasses (poaceae) and it is suggested that this genetic event preceded the evolution of the poaceae [9]. Earlier three paralogous genes of *PSY* have been identified in rice, maize and wheat [10]. Although the *PSY* genes with putative homologs have been reported in bread wheat [11] but so far their detailed expression and functional characterization have not been performed.

Efforts have been made earlier to enhance carotenoid content through transgenic approach in wheat by using *CrtB* gene [12–13] which is a homolog of *PSY* in bacteria. However, *PSY* gene isolated from plants has not been utilized for this purpose in wheat. Therefore, understanding the role of *PSY* genes in wheat and their functional characterization for carotenoid accumulation could be the step forward in the direction to identify candidate genes for the development of genetically improved wheat crop. In the current study, nine putative wheat *PSY* (*TaPSY*) sequences were *in-silico* characterized for their structural features, chromosomal and sub-cellular localization, and phylogenetic analyses. Three *PSY* genes were identified from an Indian hexaploid wheat (*Triticum aestivum* L.) variety C306. The expression analysis of three *TaPSY* genes was performed in different tissues, during seed development stages and under stress conditions. The functional characterization of TaPSY proteins was performed by colour complementation assay in *Escherichia coli*. The expression pattern of *TaPSY* was correlated with the content of β-carotene at the seed developmental stages. The present analysis revealed that *TaPSY* can be a potential target for the modulation of β-carotene biosynthesis in wheat.

## Materials and Methods

### Identification and cloning of *TaPSY* genes

The gene model sequences of *T*. *aestivum* (cv. Chinese Spring) were downloaded from International Wheat Genome Sequencing Consortium (IWGSC) database available at the web portal (http://www.wheatgenome.org) and accessed in July 2014. A local sequence database was prepared using the National Center for Biotechnology Information (NCBI) BLAST program. PSY protein sequences of *Arabidopsis thaliana* (NP_197225.1) and *Oryza sativa* (NP_001058647.1, NP_001067325.1) were used for TBLASTN analysis against the local *T*. *aestivum* gene model sequence database to identify similar sequences in wheat. The reported *PSY* gene sequences (EF600063, BT009537) of wheat were also considered in BLAST analysis. The most similar sequences were retrieved and confirmed by BLASTX program at the NCBI (http://www.ncbi.nlm.nih.gov) and designated them as putative *TaPSY* genes. The homoeologs of *PSY* were identified on the basis of similarity between sequences and *T*. *aestivum* unigenes. The identified *TaPSY* genes were amplified from Indian wheat variety C306 by using gene specific end primers (S1 Table). These genes were cloned in pBluescript SK+ vector and confirmed by sequencing.

### *In-silico* analysis of *TaPSY* sequences

The open reading frame (ORF) of the *TaPSY* genes from cv. Chinese Spring was obtained using NCBI ORF finder (http://www.ncbi.nlm.nih.gov/gorf/gorf.html). Multiple sequence alignment was performed by using CLUSTALW program to determine the similarity between various sequences. cDNA and genomic sequences were aligned for the prediction of exon and intron. The sub-cellular localization of TaPSY proteins was predicted with PSORT server (http://psort.hgc.jp) and ProtComp 9.0 program (http://linux1.softberry.com/berry.phtml?topic=protcompan&group=help&subgroup=proloc). Prediction of signal peptides for protein secretion was performed with SignalP4.1 (http://www.cbs.dtu.dk/services/SignalP). Theoretical molecular weight (MW) and isoelectronic point (pI) of the TaPSY proteins were computed using ExPASy proteomics server (http://web.expasy.org/compute_pi). The presence of transmembrane domains was analyzed with TMHMM Server v. 2.0 (http://www.cbs.dtu.dk/services/TMHMM). The domain architecture and active sites for PSY activity were identified by using ScanProsite tool (http://prosite.expasy.org/scanprosite) and SMART server (http://smart.embl-heidelberg.de).

To analyse the *cis*-regulatory elements of *TaPSY* genes, about 1500 bp upstream promoter regions were obtained by BLASTN search of cDNA sequences against the *T*. *aestivum* chromosome sequences (https://urgi.versailles.inra.fr/blast/blast.php). The retrieved sequences were placed in the PlantCARE database (http://bioinformatics.psb.ugent.be/webtools/plantcare/html) in which a brief description of motifs was extracted.

### Phylogenetic analysis

The phylogenetic relationship of TaPSY with the known sequences of several monocots i.e., *O*. *sativa* (PSY1, Q6KBZ6; PSY2, Q6ED35; PSY3, B6UV92), *Triticum dicoccoides* (PSY1, ACQ59135.1; PSY1, ACQ59141.1), *Triticum urartu* (PSY1, ACQ59127.1; PSY2, EMS46763.1; PSY3, EMS66563.1), *Triticum monococcum* (PSY1, ACQ59129.1), *Triticum turgidum* (PSY1, ACO07290.1; PSY1, ABW80611.1; PSY2, A2T2L0), *Aegilops tauschii* (PSY1, ACY68563.1; PSY2, EMT07172.1; PSY3, EMT04591.1), *Aegilops speltoides* (PSY1, ACQ59147.1), *Hordeum vulgare* (PSY1, M0YGH3; PSY2, M0WD98), *Brachypodium distachyon* (PSY1, I1GV47; PSY2, I1IG83), and *Zea mays* (PSY1, D1GIY3; PSY2, K7V3J4; PSY3, B0KYU8) was performed. The squalene synthase sequence of *Ae*. *tauschii* (EMT03383.1) was used as outlier. The aligned regions were extracted and evolutionary history was inferred by using the Neighbor-Joining method and tree was constructed with a 1000-bootstrap replication support using MEGA 6 software [14].

### RNA isolation, cDNA synthesis and quantitative real-time PCR

Bread wheat variety C306 was grown in the experimental field of the National Agri-Food Biotechnology Institute, Mohali, Punjab, India (310 m above sea level; Latitude 30° 47’ North; Longitude 76° 41’ East). Seed samples from different developmental stages (7, 14, 21, and 28 days after anthesis; DAA), various portions of seed at 14 DAA (aleurone, embryo, endosperm, and pericarp), and tissues such as stem, leaf, flag leaf, and root were collected, frozen in liquid nitrogen, and stored at −80°C until further use. For stress treatments, germinated seedlings were subjected to heat (40°C), drought (20% (w/v) PEG-6000) and combination of both the treatments for 1 h and 6 h, as described previously [15]. Leaves were collected, frozen immediately in the liquid nitrogen, and stored at −80°C till further use. Three biological replicates for each sample were considered for the RNA isolation and real-time PCR analysis. Total RNA was isolated using Spectrum™ Plant Total RNA kit (Sigma-Aldrich,USA). DNA contamination was removed by On-column DNase I Digest set (Sigma-Aldrich, USA). The integrity and size distribution of total RNA was analyzed on 1.5% agarose gel by ethidium bromide staining. NanoQuant (Infinite®200 PRO NanoQuant, Austria) was used for quantification of RNA. The cDNA synthesis was performed using SuperScript®III First-Strand Synthesis SuperMix (Invitrogen™, USA) from 3 μg of DNA-free total RNA by following the manufacturer's instructions. The conserved sequence from A, B and D subgenomes of wheat was selected to design the primers and used for the expression analysis of *TaPSY* genes (S2 Table). The quantitative real-time PCR was performed by following SYBR Green (QuantiFast^TM^ SYBR Green PCR kit, QIAGEN) chemistry at ABI PRISM 7500 Fast Real-Time PCR System (Applied Biosystems, USA). Each reaction (10 μl) was consisted of cDNA (1 μl), gene specific primers (1 pmol each) and 2X SYBR Green PCR Master Mix (5 μl). Ct values were normalized against wheat *ADP ribosylation factor (ARF)* as its expression was shown to be consistent in different tissues [16]. The relative fold expression was calculated by using 2^-ΔΔCT^ method [17]. The results were analyzed statistically by mean of relative fold expression of transcript ± standard deviation (SD). One-way analysis of variance (ANOVA) followed by Dunnett's multiple comparison test was used to determine significant (P ≤ 0.05) difference.

### Quantification of β-carotene in wheat grains

Wheat variety C306 grains from various developmental stages were collected in two to three biological replicates and crushed with liquid nitrogen. Carotenoid was extracted following the method [18] with some modifications. In brief, the powder of sample was homogenized in 25 ml solution containing 40% aqueous methanol and 0.5 g basic magnesium carbonate. The mixture was kept in incubator shaker at 200 rpm for 30 min and then centrifuged at 8000 rpm for 15 min. The supernatant was discarded and pellet was extracted in 10 ml solution of diethyl ether and methanol (7:3, v/v) containing 0.1% (w/v) butylated hydroxy toluene (BHT) till the extract turn out to be colourless. The extract was taken in a separating funnel and 10 ml diethyl ether was added. The ether phase was washed twice with saturated sodium chloride (NaCl) and anhydrous sodium sulphate (Na_2_SO_4_) solution (2:1, v/v). The sample was evaporated using Rota Vapour at 50°C at 100 rpm and 200 atmospheric pressures. Sample was dissolved in tetrahydrofuran (THF), centrifuged and supernatant was collected. The supernatant was filtered through 0.45-μm nylon membrane filter (Millipore, MA) and was used for High Performance Liquid Chromatography (HPLC) analysis. Analysis was performed in a liquid chromatograph binary gradient module pumps (Waters, Milford, MA, USA) equipped with a photodiode array (Waters, 2998) and autosampler (Waters, 2767). The mobile phase was a gradient prepared from 95% (v/v) methanol in HPLC-grade water (component A) and methyl tertiary-butyl ether (MTBE) (component B). The standard of β-carotene (Sigma, USA) was used for calibration curve, comparison of retention time, and quantification of samples.

### Functional characterization of *TaPSY* genes in *E*. *coli*

The functional activity of *TaPSY* genes was determined by using bacterial complementation assay. The plasmid pAC-BETA provided by Dr. Francis X. Cunningham Jr, Department of Cell Biology and Molecular Genetics, University of Maryland, USA was used in this study [19]. The *E*. *coli* cells containing pAC-BETA plasmid produce and accumulate β-carotene, resulting in yellow colonies. The pBluescript SK^+^ plasmid comprising ORF of the *TaPSY1****_****7BL*, *TaPSY2_5BS*, and *TaPSY3_5DL* was individually co-transformed with pAC-BETA plasmid in *E*. *coli*. Positive colonies were selected on the Luria Agar (LA) medium containing ampicillin (100 μg/ml) and chloramphenicol (50 μg/ml) antibiotics. The empty SK^+^ plasmid was also co-transformed with pAC-BETA as negative control. The relative activity of TaPSY proteins could be observed as variation in the colour of cultures due to differential accumulation of carotenoid. To quantify the content of β-carotene, 100 ml cultures of transformed *E*. *coli* cells were grown in Luria Broth medium for 72 h at 28°C in dark. Carotenoids were extracted as explained earlier [18], and β-carotene content was analyzed using HPLC.

## Results and Discussion

### Identification of *TaPSY* genes

Blast search revealed the existence of nine putative *PSY* sequences (Traes_7AL_5262BD5AE.1, Traes_7BL_188975B53.1, Traes_7DL_BDE14D8F9.1, Traes_5BS_87F876396.1, Traes_5DS_EAD24AD6B.1, Traes_5AS_7158B51F3.1, Traes_5AL_4A55EC2ED.1, Traes_5BL_667D47F86.1, and Traes_5DL_514F6294B.1) in wheat genome database. Six of them were full length, while rest of three (Traes_7AL_5262BD5AE.1, Traes_7BL_188975B53.1, and Traes_5AL_4A55EC2ED.1) were incomplete encoding sequences. This might be due to the unavailability of complete wheat genome sequence. These three sequences were explored for full length by BLASTN search against the transcriptome shotgun assembly (TSA) sequences of *T*. *aestivum* (S1 File). All nine sequences were clustered into three groups. Each group consisted of three sequences on the basis of their similarity with each other and with *T*. *aestivum* unigenes (S3 Table). Each group consisted of one sequence from every subgenome (A, B, and D) and showed higher similarity with each other in comparison to other group (S3 Table). Therefore, these clustered sequences were considered as homoeologs. Thus, results indicated the presence of three *PSY* genes (*TaPSY1*, *TaPSY2*, and *TaPSY3*) in bread wheat with three homoeologs for each gene. Multiple copies of *PSY* are reported in several plant species [9,20,21]. Previously, many *PSY* were identified and functionally characterized that include from banana [21], tomato [22], sorghum [23], and sweet osmanthus [24]. Similar to our study, three *PSY* genes are reported in other monocots, like rice and maize [9,13].

### *In-silico* analysis of *TaPSY* genes

BLASTN search against gene model sequences has indicated that the *TaPSY1*, *TaPSY2*, and *TaPSY3* were localized on long arm of group 7 chromosome (7AL, 7BL, and 7DL), short arm of group 5 chromosome (5AS, 5BS, and 5DS), and long arm of group 5 chromosome (5AL, 5BL, and 5DL), respectively in each subgenome. Earlier, three *PSY* genes in *T*. *aestivum* have been reported but their homoeologs were not identified [10]. We noticed similar distribution pattern of *TaPSY* as reported previously [11] and incorporated additional information about *TaPSY1* homoeolog located at 7DL. Though, the ORFs of isolated *TaPSY* genes were of variable length (Table 1), but their homoeologs were quite similar in size. We observed nearly 90% similarity between homoeologous sequences of each *TaPSY* gene, however similarity between the sequences of three *TaPSY* genes varies from 57% to 67% (S3 Table, S2A, S2B and S2C File). Variable length of *PSY* genes were also reported in other monocot species like rice and maize [10]. Structural analysis of exons and introns of all the nine *TaPSY* sequences are presented in Table 1. The *TaPSY1*, *TaPSY2* and *TaPSY3* genes contained variable numbers of exons (6, 5, and 4) and introns (5, 4, and 3), respectively. In case of rice, all three *PSY* genes comprised 6 exons and 5 introns [9]. The loss of introns noticed in wheat, and previously in sorghum, and brachypodium might be governed by the fusion of exons during evolution [10]. The TaPSY proteins were variable in their length, MW and pI (Table 1). The average length (~428 AA) and MW (~47 kDa) of TaPSY1 was larger than TaPSY3 (~405 AA and ~44 kDa), followed by TaPSY2 (~280 AA and ~32 kDa). We observed more than 90% similarity between the homoeologous protein sequences of each TaPSY. However, similarity varied from 57% to 76% between the groups (S4 Table, S3A, S3B and S3C File).

**Table 1:** *In-silico* characterization of *TaPSY* genes.

Gene	Homeologs	Unigene	ORF length (bp)	Exon (Intron)	Protein length (AA)	MW (kDa)/pI	Cellular location	Signal peptide	Transmembrane region
*TaPSY*1									
	*PSY1_7DL* *PSY1_7BL* *PSY1_7AL*	Ta.20776 Ta.20776 Ta.20776	1293 1275 1296	6(5) 6(5) 6(5)	430 424 431	48/9.01 47/9.13 47/8.96	Chloroplast Chloroplast Chloroplast	P P P	P P P
*TaPSY*2									
	*PSY2_5BS* *PSY2_5AS* *PSY2_5DS*	Ta.41960 Ta.41960 Ta.41960	846 843 846	5(4) 5(4) 5(4)	281 280 281	32/8.37 32/7.65 32/8.29	Chloroplast Chloroplast Chloroplast	P P P	P P P
*TaPSY*3									
	*PSY3_5DL* *PSY3_5AL* *PSY3_5BL*	Ta.117209 Ta.117209 Ta.117209	1212 1242 1206	4(3) 4(3) 4(3)	403 413 401	44/9.24 45/9.09 43/9.25	Chloroplast Chloroplast Chloroplast	ND ND ND	ND ND ND

P: Present, ND: Not detected

### Functional domain analysis

**Fig 1:**
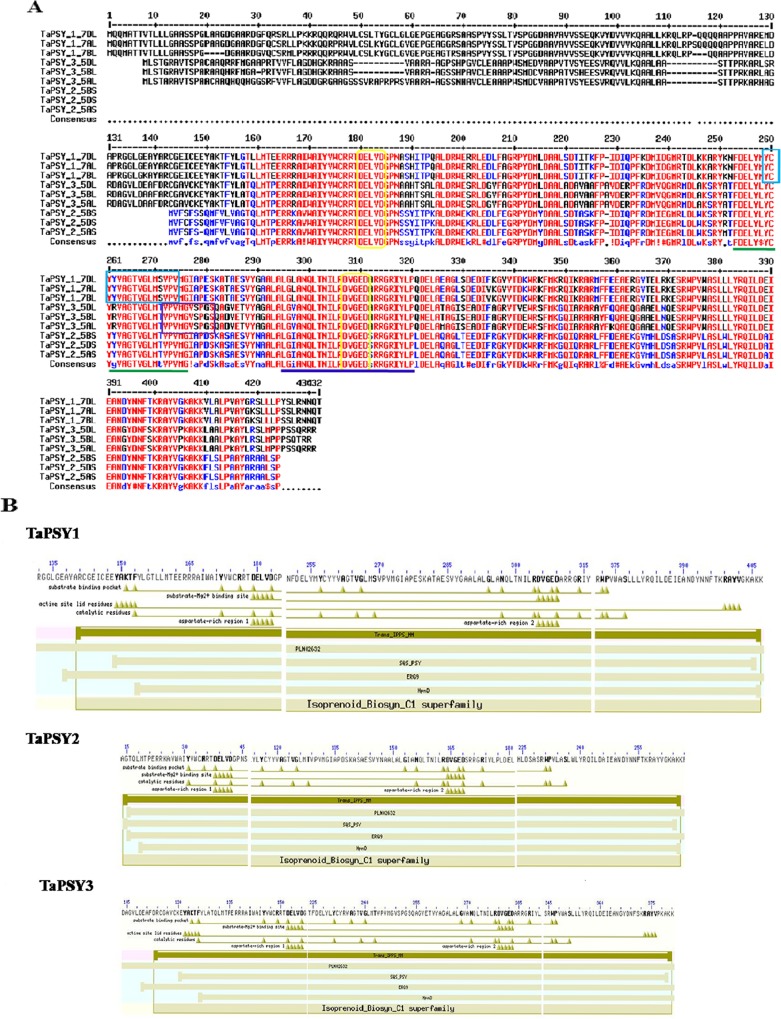
Multiple sequence alignment and conserved domains analysis of TaPSY from cv. Chinese Spring. **(A)** Alignment shows that the amino acids at C-terminal region are highly conserved in all TaPSY. Green and blue underlines indicate the positions of trans-membrane helix and squalene/phytoene synthase signature 2 “LGlanQlt.NIlRDVgeDarrg…RiYlP” (PS01045), respectively. Light blue and purple colour boxes show motifs for squalene/phytoene synthase signature 1 “YCyyVAGTVGlmSvpV” (PS01044) in PSY1 and lipases serine active site “VPVMGVSPGS” (PS00120) in PSY3, respectively. Yellow colour boxes indicate the positions of putative PSY active sites (DXXXD). **(B)** Conserved domains in TaPSY are detected by NCBI conserved domain BLAST search analysis. Trans-isoprenyl diphosphate synthases (trans-IPPS_HH) (cd00867) domain consist of two putative aspartate rich regions (DELVD and DVGED) and substrate-Mg^2+^ binding sites are detected in all TaPSY. Two active site lid residues (YAKTF and RAYV) are presented in TaPSY1 and TaPSY3, while one (RAYV) is found in TaPSY2.

The TaPSY proteins were highly conserved at the C-terminus and variable at the N-terminus (Fig 1A). The domain architecture analysis indicated the presence of squalene/phytoene synthase signature 2 “LGlanQlt.NIlRDVgeDarrg…RiYlP” (PS01045) motif in the TaPSY1, TaPSY2 and TaPSY3. However, squalene/phytoene synthase signature 1 “YCyyVAGTVGlmSvpV” (PS01044) and lipases serine active site “VPVMGVSPGS” (PS00120) were present in the TaPSY1 and TaPSY3, respectively (Fig 1A and 1B). The serine residue was absent in lipases serine active site of TaPSY1 and TaPSY2 proteins. The conserved trans-isoprenyl diphosphate synthases, head-to-head (trans-IPPS_HH) domain catalyzes the production of phytoene by condensation of two molecules of GGPP. The analyses revealed that a conserved trans-IPPS_HH domain (cd00867), two putative PSY active sites (DELVD and DVGED), aspartate-rich motif (ARM) and Mg^2+^ binding sites were found in each TaPSY protein. Two active site lid residues (YAKTF and RAYV) were detected in TaPSY1 and TaPSY3, while one (RAYV) was present in TaPSY2 (Fig 1B). These conserved domains and sites are also reported in other plant species, indicating their role in PSY functional activity [10,25].

The TaPSY proteins were predicted to be generally localized in the chloroplast (Table 1). Since, carotenoids are predominantly synthesized through plastid localized 2-C-methyl-D-erythritol 4-phosphate (MEP) pathway in chloroplast, it is anticipated that the nuclear encoded enzymes involved in this pathway should be localized in the same organelle [8]. A signal peptide was detected in TaPSY1 and TaPSY2. The PSY proteins are considered as membrane protein in various studies [26,27]. A transmembrane domain was predicted in TaPSY1 and TaPSY2 proteins, supporting their possible membrane-bound nature. In contrast, no signal peptide and transmenbrane region were predicted in the TaPSY3 protein. Although, the alignment of protein sequences indicated the presence of these features in TaPSY3 (Fig 1A), which might be responsible for their chloroplastic localization. These observations are based on *in-silico* analysis and further need to be validated for actual localization of the proteins.

### Promoter analysis

The identified *cis*-regulatory motifs present in the promoter region of *TaPSY* genes were categorized in relation to development, light, and stress responses (Table 1). Diversity in the occurrence of *cis*-regulatory elements was observed in *TaPSY* genes, however Skn-1_motif, G-Box, I-Box, and ABRE elements were present in all three genes. Similar motifs were earlier reported in other plant species like rice and sorghum [28,29]. We noted that the growth and development related motifs were mostly localized on the promoters of *TaPSY1* and *TaPSY2*. Higher number of motifs related to stress response were found at *TaPSY3*, that indicated their role during stress conditions. Similarly, rice *PSY3* is also reported to be involved in abiotic stress [28]. The ABRE element was detected in all *TaPSY*, in contrast to its sole presence on *PSY3* in rice and maize [30]. Various other regulatory elements such as O2-site, Skn-1_motif, CCGTCC-box, and GCN4_motif related to growth and development, GATA, TCCC, GC, CATT, and GAG motifs related to light response, and TGACG, GARE, MBS, W-Box, LTR, and CGTCA motifs related to stress responses were also identified. The diversity in the occurrence of *cis*-regulatory elements in *TaPSY* genes has indicated their possible role during development and stress conditions.

### Phylogenetic relationship

**Fig 2:**
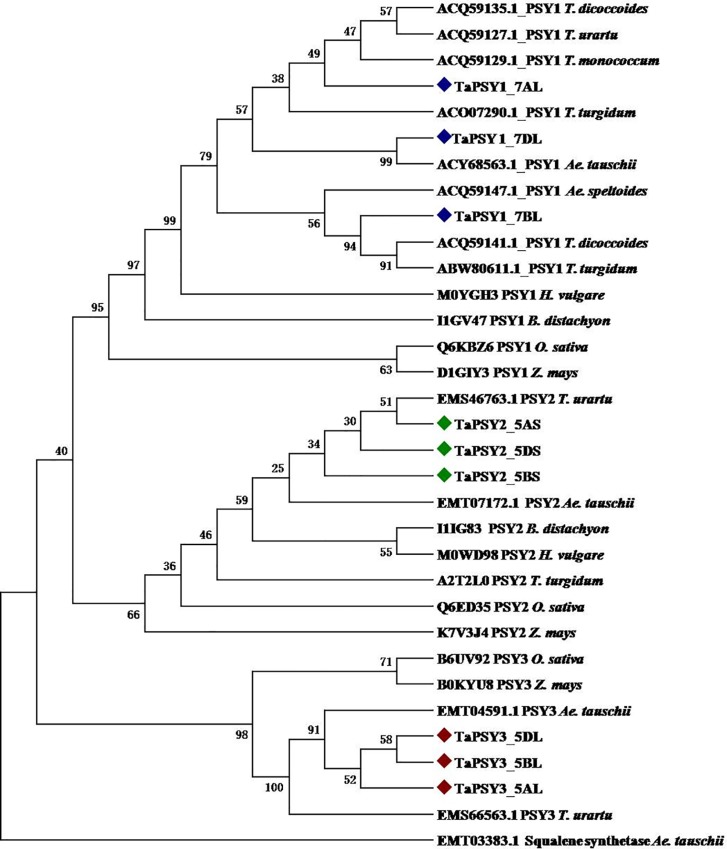
Phylogenetic tree analysis of the PSY protein sequences. A Maximum Parsimony based phylogenetic tree of the wheat and other monocots was generated by using MEGA 6 software. The sequences of *O*. *sativa (*PSY1, Q6KBZ6; PSY2, Q6ED35; PSY3, B6UV92), *T*. *dicoccoides* (PSY1, ACQ59135.1; PSY1, ACQ59141.1), *T*. *urartu* (PSY1, ACQ59127.1; PSY2, EMS46763.1; PSY3, EMS66563.1), *T*. *monococcum* (PSY, ACQ59129.1), *T*. *turgidum* (PSY1, ACO07290.1; PSY1, ABW80611.1; PSY2, A2T2L0), *Ae*. *tauschii* (PSY1, ACY68563.1; PSY2, EMT07172.1; PSY3 EMT04591.1), *Ae*. *speltoides* (PSY1, ACQ59147.1), *H*. *vulgare* (PSY1, M0YGH3; PSY2, M0WD98), *B*. *distachyon* (PSY1, I1GV47; PSY2, I1IG83), and *Z*. *mays* (PSY1, D1GIY3; PSY2, K7V3J4; PSY3, B0KYU8) were considered. The squalene synthase sequence of *Ae*. *tauschii* (EMT03383.1) was used as outlier. The sequences were aligned using MUSCLE and the phylogenetic tree was created by Neighbor-Joining method with 1000 bootstrap replicates using MEGA 6 software. The numbers shown at each node represent bootstrap values.

The evolutionary relationship was inferred using 33 different PSY sequences from various monocot species. Result showed the categorization of PSY sequences in three groups for PSY1, PSY2 and PSY3 (Fig 2). As expected, the TaPSY homoeologous sequences were clustered together within their related groups. The similar phylogenetic relationship was also reported earlier in other studies [9,25]. The homoeologous sequences for each TaPSY were localized in tree as per their genomic position. The TaPSY derived from A and D subgenomes were found in the close vicinity of the PSY sequences of *T*. *urartu* and *Ae*. *Tauschii*, which is due to the contribution of A and D subgenomes in bread wheat (*T*. *aestivum*, AABBDD) from these two species [31–32].

### Expression analysis of *TaPSY* genes

**Fig 3:**
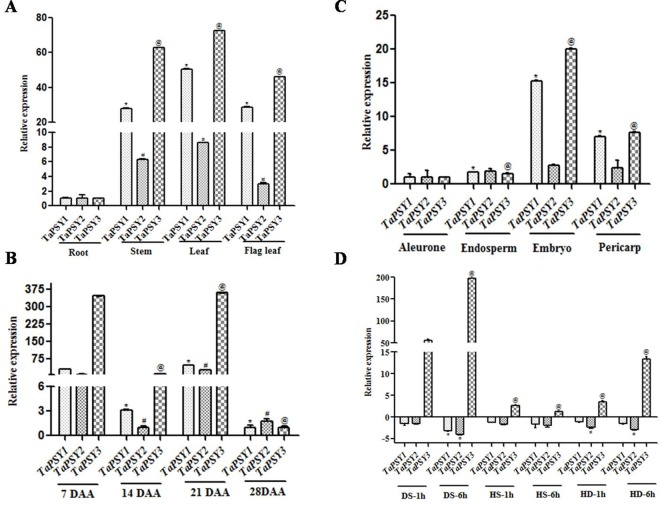
Tissue specific real-time PCR expression analysis of *TaPSY1*, *TaPSY2* and *TaPSY3* genes in wheat. **(A)** Transcript **e**xpression patterns in root, stem, leaf, and flag leaf. **(B)** Expression during different seed developmental stages. **(C)** Quantification of transcripts in various layers (aleurone and pericarp) and tissues (endosperm and embryo) of wheat seeds. **(D)** Expression of *TaPSY* genes in leaves when wheat plants exposed with drought stress (DS), heat stress (HS), and combined of both heat and drought (HD) stresses for 1 h and 6 h treatment. The level of all TaPSY transcripts normalized with reference to *ARF* taken as an internal control. The transcripts fold expression was calculated relative to the lowest expressing tissue for each gene in each experiment. All values represent mean ± SD. One-way analysis of variance (ANOVA) using Dunnett's multiple comparison test was performed to check the level of significance. Statistical significance (p ≤ 0.05) was analyzed with respect to the expression of each gene in root or 7 DAA or aleurone or DS-1h (drought stress for 1 h treatment) in the respective experiments. In graphs the symbols denoted with *, #, and @ represent significant difference at p ≤ 0.05 for the *TaPSY1*, *TaPSY2*, and *TaPSY3* respectively in the experiments.

The expression pattern of *TaPSY* genes was analyzed in different tissues and various developmental stages of wheat grain by quantitative real-time PCR (Fig 3). The lower expression of all *TaPSY* genes was noticed in root as compared to the other green tissues. The expression of *TaPSY1* and *TaPSY3* was significantly higher than *TaPSY2* in stem, leaf, and flag leaf (Fig 3A). The promoter analysis showed the presence of light responsive motifs in *TaPSY* genes. Light induced expression of carotenoid biosynthesis pathway genes are earlier reported in several studies [33–34]. The regulatory effects of light in *PSY* expression has been reported to modulate carotenoid accumulation in various plant species [34,35,36]. Thus the presence of light responsive elements in *TaPSY* promoters might be responsible for their higher expression in green tissues as compared to the root. In contrast, the significant down regulation of carotenoid biosynthetic genes was observed during the dark condition [33–35]. Expression analysis in different developmental stages of wheat grain showed an interesting trend. Higher *TaPSY* expression was observed at 7 and 21 DAA than 14 DAA. Lowest expression detected at late seed development stage i.e., 28 DAA, could be accounted due to the maturation of grain. The expression of *TaPSY3* was higher at 7, 14, and 21 DAA, while *TaPSY1*, and *TaPSY2* showed nearly similar expression except at 14 DAA (Fig 3B). The higher expression of *TaPSY3* might be related with their inducible nature during drought stress. Similar drought inducible expression of *PSY3* was reported in rice seed [28] as the wheat grains usually countenance similar conditions. The expression of *TaPSY* genes was also analyzed in various layers (pericarp, aleurone), endosperm and embryo of wheat grain at 14 DAA. The overall expression of all *TaPSY* genes was low but almost similar to that in aleurone and endosperm. *TaPSY2* expression was nearly similar in all the seed tissues and layers. The highest expression of *TaPSY1* and *TaPSY3* was observed in embryo, followed by pericarp. The expression of *TaPSY3* was higher than *TaPSY1* in both embryo and pericarp (Fig 3C). Variation in gene expression between the paralogs in duplicated genomes can be a consequence of the polyploidization or a result of changes introduced over the time period in the genome [37].

Several putative *cis*-acting elements selectively involved in abiotic stress response were present in the proximal promoter regions of *TaPSY* genes (Table 2). The presence of these elements in PSY of different plant species have been reported to modulate their expression [28,29,38]. Therefore, we carried out expression analysis under the heat, drought and combination of both stresses to observe the modulation in expression pattern of *TaPSY* genes. The *TaPSY3* was found upregulated during each stress condition but at different extent, while *TaPSY1* and *TaPSY2* were downregulated. The *TaPSY3* was highly upregulated during the drought stress. The transcript fold accumulation of *TaPSY3* during drought stress was observed upto 55- and 196-folds after 1 and at 6 h of treatment, respectively (Fig 3D). These results are in agreement with previous studies where the role of *PSY3* is reported during drought stress in rice [28] and maize [29] with the presence of higher number of stress related *cis*-regulatory elements. The results conclude conserved role of the *PSY3* for its involvement in drought response in wheat. The similar observation was reported earlier in other plant species [10,30].

**Table 2:** *Cis*-regulatory elements found in the promoter region of *TaPSY* genes.

**Motifs related to growth and development**			
	*TaPSY1*	*TaPSY2*	*TaPSY3*
GCN4_motif	+	-	-
O2-site	+	+	-
Skn-1_motif	+	+	+
CCGTCC-box	-	+	_
**Motifs related to light response**			
Box 4	+	+	_
GATA-motif	+	-	-
TCCC-motif	+	-	-
G-Box	+	+	+
I-box	+	+	+
GC- motif	+	-	+
CATT-motif	+	+	-
GAG-motif	-	+	+
GT1-motif	-	+	-
Sp1	-	-	+
ACE	-	-	+
**Motifs related to stress response**			
ABRE	+	+	+
TGACG-motif	+	-	+
GARE-motif	+	-	+
motif IIb	+	-	-
MBS	-	-	+
W box	-	-	+
LTR	-	-	+
CGTCA-motif	-	-	+

+: Present,—: Absent

### Analysis of β-carotene content in wheat grain

**Fig 4:**
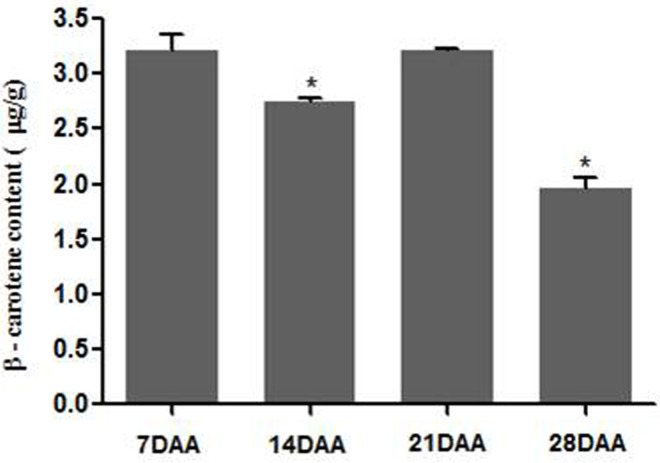
Estimation of β-carotene content in developing grains of wheat at different stages. Analysis was performed by HPLC. Each bar indicates the mean of two to three replicates ±SD. Statistical significance was checked by using one-way analysis of variance (ANOVA) at p ≤ 0.05 denoted as *, with respect to the content in 7 DAA.

The β-carotene content of wheat grain was analyzed by HPLC at different developmental stages (Fig 4). It was highest and similar at 7 and 21 DAA, which was followed by 14 and 28 DAA. The lower β-carotene was detected at 28 DAA among all developmental stages. Though the β-carotene accumulation was reported in matured grain of wheat [39], but this is the first report that demonstrated the pattern of β-carotene biosynthesis in various developmental stages of wheat grain. To certain extent, the correlation was observed between the accumulation of β-carotene content with the expression of *TaPSY* genes during various developmental stages of grains (Fig 3B). Since the PSY is a rate limiting enzyme in carotenoid biosynthetic pathway, their expression pattern may also affect the accumulation of β-carotene [6]. Based on this work, we speculate that the overexpression of *TaPSY3* genes in wheat grain may increase the β-carotene content and could also provide resistance against the abiotic stress. Although *PSY* transcripts could not directly be correlated with the content of carotenoid levels [40]. This has suggested that post-transcriptional mechanism may have an important role for determining flux through this enzymatic step. Hence, it would be interesting to validate the functional activity of TaPSY3 protein in other model plant species like arabidospsis.

### Functional analysis of *TaPSY* by complementation assay

**Fig 5:**
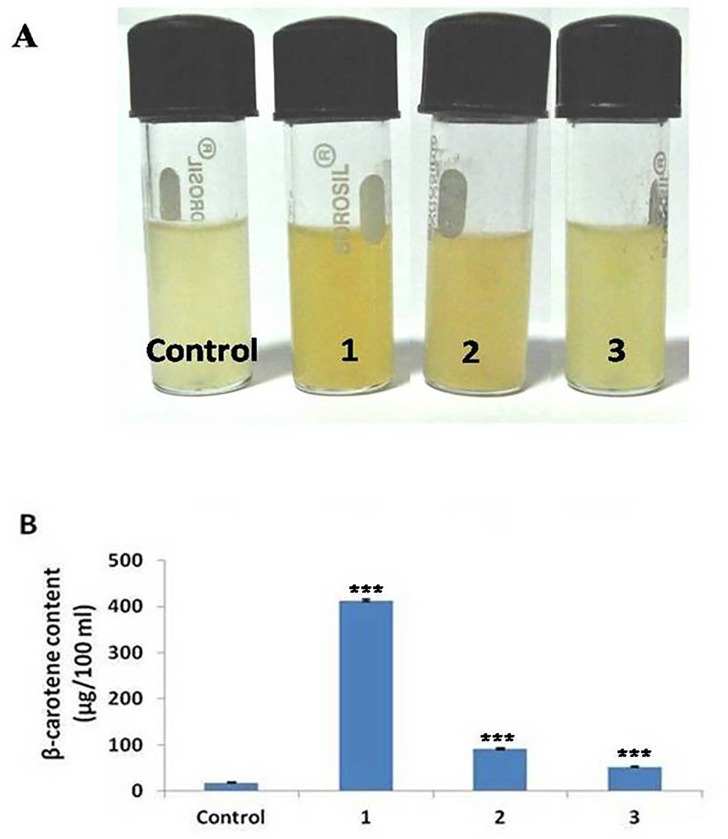
Functional complementation assay. **(A)** Visual observation of *E*. *coli* suspension cultures consisting of pAC-BETA and complemented with plasmids containing *TaPSY* genes. **(B)** Concentration of β-carotene in *E*. *coli* cells after complementation with *TaPSY* genes. The Control represents for pAC-BETA + empty SK^+^ while 1, 2 and 3 represent complementation with plasmids containing pAC-BETA + *TaPSY1*(SK^+^), pAC-BETA + *TaPSY2*(SK^+^) and pAC-BETA + *TaPSY3*(SK^+^), respectively. Statistical significance was checked by using one-way analysis of variance (ANOVA) at p ≤ 0.001 (highly significant) and denoted as ***, with respect to the Control.

A functional colour complementation approach is earlier reported to be used to analyze the activity of genes/enzymes involved in carotenoid pathway in *E*. *coli* [8,19]. The co-transformed *E*. *coli* cells producing β-carotene as the end product of the pathway, which could be monitored by the change in colour of bacterial cultures (Fig 5). The intense yellow colour was observed in *TaPSY1* containing recombinant *E*. *coli* culture, which was followed by *TaPSY2* and *TaPSY3* (Fig 5A). The results indicated that all the TaPSY enzymes were functionally active and involved in carotenoid biosynthesis in recombinant *E*. *coli* cells. However, variation in colour composition indicated differences in their activity. This approach has been utilized for the functional characterization of carotenoid pathway genes of various plants including apple [40] and grape [41]. We found similar trend of β-carotene content by HPLC analysis as noticed in colour change of bacterial cultures (Fig 5A and 5B). The highest β-carotene content was detected in *TaPSY1* containing cells, which was followed by *TaPSY2* and *TaPSY3*. These results established that all the identified *TaPSY* genes were enzymatically active and could accelerate the biosynthesis of β-carotene at different extent. These results indicated that TaPSY1 enzyme was more active in bacterial system. However, it could be the result of variety of factors including catalytic activity of enzymes, protein localization, stability, folding, solubility and differences in amino acid sequences between different genes.

## Conclusions

The PSY is an important rate limiting enzyme in carotenoid biosynthetic pathway. Present study identified three *PSY* genes (*TaPSY1*, *TaPSY2 and TaPSY3*) each with three homoeologs in wheat. Since the carotenoid biosynthesis occurs in chloroplast, TaPSY proteins were predicted to be localized in the same organelle. Analysis of *cis*-regulatory elements has indicated diverse role of various *TaPSY* genes which is supported by the differential expression pattern of these genes during different development and stress conditions of plants. The colour complementation assay indicated higher activity of the *TaPSY1* gene in β-carotene synthesis, while the *TaPSY3* was more active during the expression analysis in different tissues, seed developmental stages and stress responses. The carotenoid content in grain is earlier correlated with the flour colour of wheat [11]. They found that the flour colour is determined by the activities of *TaPSY1* and *catalase* genes located on the long arm of chromosome 7 in A subgenome. We also noted that the *TaPSY1* was more active in carotenoid accumulation during bacterial complementation assay. Though, it remains to be seen if other carotenoid degrading enzymes commonly known as carotenoid cleavage dioxygenases (CCDs), also play critical role in carotenoid accumulation in wheat grain.

## Supporting Information

S1 File Full length sequences of *TaPSY*1 (7DL, 7BL, and 7AL), *TaPSY*2 (5BS, 5DS, and 5AS), and *TaPSY3* (5DL, 5AL, and 5BL) genes. (DOC)

S2 File Alignment and similarity analysis of identified *TaPSY* genes encoded by homoeologous sequences from A, B and D subgenomes of *T*. *aestivum*. (A) Alignment of *TaPSY1* located on the long arm of group 7 chromosome. Percent identity between the sequences located on 7A:7B, 7A:7D, and 7B:7D are 97%, 94%, and 90%, respectively. (B) Alignment of *TaPSY*2 located on the short arm of group 5 chromosome. Percent identity between the sequences located on 5A:5B, 5A:5D, and 5B:5D are 95%, 96%, and 96%, respectively. (C) Alignment of *TaPSY*3 located on the long arm of group 5 chromosome. Percent identity between the sequences located on 5A:5B, 5A:5D, and 5B:5D are 89%, 92%, and 95%, respectively.
(DOC)

S3 File Alignment and similarity analysis of amino acid sequences of TaPSY proteins encoded by homoeologous sequences from A, B, and D subgenomes of *T*. *aestivum*. (A) Alignment of TaPSY1 located on the long arm of group 7 chromosome. Percent identity between 7A:7B, 7A:7D, and 7B:7D are 96%, 96%, and 95%, respectively. (B) Alignment of TaPSY2 located on the short arm of group 5 chromosome. Percent identity between 5A:5B, 5A:5D, and 5B:5D are 98%, 97%, and 99%, respectively. (C) Alignment of TaPSY3 located on the long arm of group 5 chromosome. Percent identity between 5A:5B, 5A:5D, and 5B:5D are 93%, 91%, and 95%, respectively.
(DOC)

S1 Table List of primers used for the amplification of *TaPSY* genes. (DOCX)

S2 Table List of primers used for the quantitative real-time PCR analysis of *TaPSY* genes. (DOCX)

S3 Table Similarity analysis between identified *TaPSY* gene sequences. (DOCX)

S4 Table Similarity analysis between identified TaPSY protein sequences. (DOCX)
