# Supplementary material for: Characterization and Expression Analysis of Phytoene Synthase from Bread Wheat (Triticum aestivum L.)
Source: PLoS One. 2016 Oct 3;11(10):e0162443. doi: 10.1371/journal.pone.0162443 (PMC5047459; doi:10.1371/journal.pone.0162443)
Supplement: S1 File — (DOC) [file pone.0162443.s001.doc]

**S1 File.** Full length sequences of *TaPSY*1 (7DL, 7BL, and 7AL)*, TaPSY*2 (5BS, 5DS, and 5AS), and *TaPSY3* (5DL, 5AL, and 5BL) genes.

>*TaPSY1_7DL*

ATGCAGCAGATGGCCACCACCGTCACGCTGCTGCTCGGGGCAGCCTCGTCCCCAGCCTCGCCGCCGGTGATGGCGCCGCGCGGGACGGCTTCCAGCGCTCCCGCCTGCTGCCCAAGAAGCGGCAGCAGAGGCAACGCTGGGTGCTCTGCTCGCTCACGTACGGCTGCCTCGGCCTCGGCGAGCCGGGGGAGGCGGGCGGCCGGAGCGCGGCGTCCCCGGTGTACTCCAGCCTCACCGTCAGCCCCGGCGGCGACGCGGCCGTCGCCGTCGTCTCGTCGGAGCAGAAAGTGTACGACGTGGTGGTGAAGCAGGCGGCATTGCTCAAGCGCCAGCTGCGCCCGCAGCAGCAGCAGGCGGCGCCGCCCGCCGTCGCCAGGGAGATGGACGCGCCGCGCGGCGGGCTCGGGGAGGCCTACGCCCGCTGCGGCGAGATCTGCGAGGAGTACGCCAAGACCTTCTACCTCGGGACCTTGCTGATGACGGAGGAGCGGCGGCGCGCCATATGGGCCATCTACGTGTGGTGTAGGAGGACAGACGAGCTGGTGGACGGGCCGAACGCGTCGCACATCACGCCGCAGGCGCTGGACCGGTGGGAGAGGAGGCTGGAGGACCTCTTCGCCGGGCGCCCCTACGACATGCTCGACGCCGCGCTCTCCGACACCATCACCAAGTTCCCCATAGATATTCAGCCCTTCAAGGACATGATCGACGGGATGCGGACGGACCTTAAGAAGGCGAGGTACAAGAACTTTGACGAGCTCTACATGTACTGCTACTATGTTGCCGGCACCGTGGGGTTGATGAGCGTCCCGGTGATGGGCATTGCGCCGGAGTCCAAGGCGACAGCCGAGAGCGTCTATGGCGCCGCTCTGGCTCTTGGCCTCGCGAACCAGCTCACCAACATACTCAGGGATGTTGGAGAAGATGCAAGAAGAGGGAGGATATATTTGCCACAAGACGAGCTTGCGGAGGCGGGGCTCTCCGATGAAGACATCTTCAAAGGAGTCGTCACCGACAAGTGGAGGAAATTCATGAAGAGGCAGATCAAGAGGGCGAGGATGTTCTTCGAGGAGGCGGAGCGAGGGGTGACTGAGCTTAGGAAGGAGAGCCGGTGGCCGGTTTGGGCCTCTCTGTTGTTGTACCGGCAGATCCTCGATGAGATCGAAGCGAATGACTACAACAACTTCACCAAGAGGGCCTATGTTGGGAAGGCGAAGAAGGTGCTTGCGCTCCCTGTCGCGTACGGGAGATCGCTGCTCTTACCGTATTCACTGAGAAATAACCAGACCTAG

>*TaPSY1_7BL*

ATGCAGCAGATGGCCACCACCGTCACGCTGCTGCTCGGGGCAGCCTCGTCCCCCGGTGATGGCGCCGCGCGGGACGGCGTCCAGTGCTCCCGCATGTTGCCTAGGAGGAGGCAGCAGAGGCCGCGGTGGGTGCTCTGCTCGCTCAAGTACGGCTGCCTCGGCGTCGGCGAGCCGGGGGAGGCGGGAACCCGGAGCGCGGCGTCGCCGGTCTACTCCAGCCTCACCGTCAGCCCCGGAGGCGACGCCGCCGTCGCCGTCGTCTCGTCGGAGCAGAAGGTGTACGACGTGGTGGTGAAGCAGGCGGCATTGCTCAAGCGCCAGCTCCGGCCACAGCAGGCGGCGCCGCCCGCCGTAGCCAGGGAGCTGGACGCGCCGCGCGGTGGGCTCGGGAAGGCCTACGCCCGCTGCGGCGAGATCTGCGAGGAGTACGCCAAGACCTTCTACCTCGGGACCTTGCTGATGACAGAGGAGCGGCGACGCGCCATATGGGCCATCTACGTGTGGTGCAGGAGGACAGACGAGCTGGTGGACGGTCCCAACGCGTCGCACATCACGCCGCAGGCGCTGGACCGGTGGGAGAGGAGGCTGGAGGACCTCTTCGCCGGGCGCCCCTACGACATGCTCGACGCCGCGCTCTCCGACACCATCACCAAGTTCCCCATAGATATTCAGCCCTTCAAGGACATGATCGACGGGATGCGGACGGACCTCAAGAAGGCAAGATACAAGAACTTCGATGAGCTCTACATGTACTGCTACTACGTCGCAGGCACGGTGGGGCTGATGAGCGTCCCGGTGATGGGCATTGCGCCCGAGTCCAAGGCGACGGCTGAGAGCGTCTACGGCGCCGCTCTGGCTCTCGGGTTGGCGAACCAGCTCACCAACATACTCAGGGATGTCGGAGAAGATGCAAGAAGAGGAAGGATATATTTGCCGCAAGACGAGCTCGCGGAGGCAGGGCTCTCCGATGAAGACATCGTCAAAGGAGTCGTCACTGACAAGTGGAGGAAATTCATGAAGAGGCAGATCAAGAGGGCAAGGATGTTCTTCGAGGAGGCAGAGCGAGGGGTGACCGAGCTCAGGAAGGAGAGCCGGTGGCCGGTTTGGGCCTCTCTGTTGTTGTACCGGCAGATCCTCGATGAGATCGAAGCGAACGACTACAACAACTTCACCAAGAGGGCCTATGTTGGAAAGGCGAAGAAAGTGTTGGCGCTCCCTGTCGCATACGGGAAATCGCTGCTCTTACCGTCTTCACTGAGAAATAACCAGACCTGA

>*TaPSY1_7AL*

ATGCAGCAGATGGCCACCACCGTCACGCTGCTGCTCGGGGCAGCCTCGTCCCCCGGCCCCGCCGCCGGTGATGGCGCCGCGCGGGACGGCTTCCAGTGCTCCCGCCTGCTACCCAAGAAGAAACAGCAGAGGCCGCGCTGGGTGCTCTGCTCGCTCAAGTACGGCTGCCTCGGCGTCGGCGAGCCGGGGGAGGCGGGCGGCCGGAGCGCGGCGTCGCCGGTGTACTCCAGCCTCACCGTCAGCCCCGGAGGCGACGCCGCCGTCGCCGTCGTCTCGTCGGAGCAGAAGGTGTACGACGTGGTGGTGAAGCAGGCGGCATTGCTCAAGCGCCAGCTGCGCCCCTCGCAGCAACAGCAGCAGGCGCCGCCAGCCGTCGCCAGGGAGCTGGACGCGCCGCGCGGCGGGCTCGGGGAGGCCTACGCCCGCTGCGGCGAGATCTGCGAGGAGTACGCCAAGACCTTCTACCTCGGGACCTTGCTGATGACCGAGGAGCGGCGGCGCGCCATATGGGCCATCTACGTATGGTGCAGGAGGACAGACGAGCTGGTGGACGGTCCCAACGCGTCGCACATCACGCCGCAGGCGCTGGACCGGTGGGAGAGGAGGCTGGAGGACCTCTTCGCCGGGCGCCCCTACGACATGCTCGACGCCGCGCTCTCCGACACCATCACCAAGTTCCCCATAGATATTCAGCCCTTCAAGGACATGATCGACGGGATGCGGACGGACCTCAAGAAGGCAAGATACAAGAACTTCGATGAGCTCTACATGTACTGCTACTACGTCGCAGGCACGGTGGGGCTGATGAGCGTCCCGGTGATGGGCATTGCGCCCGAGTCCAAGGCGACGGCTGAGAGCGTCTACGGCGCCGCTCTGGCTCTCGGGTTGGCGAACCAGCTCACCAACATACTCAGGGATGTCGGAGAAGATGCAAGAAGAGGAAGGATATATTTGCCGCAAGACGAGCTCGCGGAGGCAGGGCTCTCCGATGAAGACATCGTCAAAGGAGTCGTCACTGACAAGTGGAGGAAATTCATGAAGAGGCAGATCAAGAGGGCAAGGATGTTCTTCGAGGAGGCAGAGCGAGGGGTGACCGAGCTCAGGAAGGAGAGCCGGTGGCCGGTTTGGGCCTCTCTGTTGTTGTACCGGCAGATCCTCGATGAGATCGAAGCGAACGACTACAACAACTTCACCAAGAGGGCCTATGTTGGAAAGGCGAAGAAAGTGTTGGCGCTCCCTGTCGCATACGGGAAATCGCTGCTCTTACCGTCTTCACTGAGAAATAACCAGACC TGA

>*TaPSY2_5BS*

ATGGTCTTTAGTTTCTCATCACAGATGTTTGTTCTTGTGGCAGGCACACAGCTTATGACTCCTGAAAGGCGCAAAGCTGTTTGGGCAATTTATGTATGGTGCAGGAGAACTGATGAGCTAGTGGATGGCCCTAACTCGTCTTACATCACGCCCAAGGCGCTCGATCGGTGGGAGAAGAGATTGGAGGATCTCTTCGAAGGCCGCCCGTATGATATGTATGACGCAGCCCTCTCAGACACGGCGTCAAAGTTTCCAATTGATATCCAGCCATTCAGAGACATGATTGAAGGGATGAGGCTCGACCTTTGGAAATCGAGGTATAGGACCTTTGACGAGCTTTACCTCTACTGCTACTACGTCGCTGGCACTGTCGGTCTCATGACGGTACCGGTGATGGGGATTGCTCCGGACTCAAAGGCCTCAGCAGAGAGCGTGTACAATGCCGCACTGGCCCTTGGCATTGCCAACCAGCTCACAAACATCCTCCGAGATGTAGGAGAAGACTCGAGGAGGGGGAGAATATACCTTCCACTGGACGAGCTGGCACAGGCGGGCCTGACAGAGGAGGACATATTCAGAGGGAAAGTGACGGATAAATGGAGGAGGTTCATGAAGGGCCAAATCCAGCGCGCCAGGCTCTTCTTCGATGAGGCCGAGAAGGGCGTCATGCATCTGGACTCCGCGAGCAGATGGCCGGTCCTGGCTTCGCTGTGGCTGTACAGGCAGATCCTTGACGCCATCGAGGCGAACGACTACAATAACTTCACCAAGCGTGCGTACGTGGGCAAGGCGAAGAAGTTCCTGTCGTTGCCGGCCGCGTACGCGAGGGCGGCTCTTTCGCCATGA

>*TaPSY2_5AS*

ATGGTCTTTAGTTTCTCACAGATGTTTGTTCTTGTGGCAGGCACACAGCTTATGACTCCTGAAAGGCGCAAAGCCGTTTGGGCGATCTATGTATGGTGCAGGAGAACTGATGAGCTAGTGGATGGCCCTAACTCATCTTACATCACGCCCAAGGCGCTGGATCGGTGGGAGAAGAGATTAGAGGATCTCTTCGAAGGCCGCCCATATGATATGTACGACGCAGCCCTCTCTGACACGGCGTCAAAGTTTCCAATTGATATCCAGCCGTTCAGAGATATGATTGAAGGGATGAGGCTTGACCTTTGGAAATCGAGGTATATGACCTTTGACGAGCTTTACCTCTACTGCTACTACGTCGCTGGCACTGTCGGTCTCATGACGGTACCGGTGATGGGGATTGCTCCGGACTCAAAGGCCTCAGCAGAGAGCGTGTACAATGCCGCACTGGCCCTTGGCATTGCCAACCAGCTCACAAACATCCTCCGAGATGTAGGAGAAGACTCAAGGAGGGGGAGAATATACCTTCCACTGGACGAACTGGCACAGGCGGGTCTGACAGAAGAGGACATATTCAGAGGGAAAGTGACGGATAAATGGAGGAGGTTCATGAAGGGCCAAATCCAGCGCGCCAGGCTCTTCTTTGATGAGGCCGAGAAGGGTGTCATGCATCTAGACTCTGCGAGCAGATGGCCGGTCCTGGCATCGCTGTGGCTGTACAGGCAGATCCTGGACGCCATCGAGGCGAACGACTACAACAACTTCACCAAGCGGGCTTACGTGGGCAAGGCGAAGAAGTTTCTGTCTCTACCGGCGGCGTACGCGAGGGCGGCTCTTTCGCCATGA

>*TaPSY2_5DS*

ATGGTCTTTTGTTTCTCATCCCAGATGTTTGTTTTTGTGGCAGGCACACAGCTTATGACTCCTGAAAGGCGCAAAGCCGTTTGGGCAATCTATGTATGGTGCAGGAGAACTGATGAGCTAGTGGATGGCCCTAACTCATCTTACATCACGCCCAAGGCGCTCGATCGGTGGGAGAAGAGATTAGAGGATCTCTTCGAAGGCCGCCCATATGATATGTATGATGCAGCCCTCTCAGATACAGCGTCAAAGTTTCCAATTGATATCCAGCCATTCAGAGACATGATTGAAGGGATGAGGCTCGACCTTTGGAAATCGAGGTATAGGACCTTTGACGAGCTCTACCTCTACTGCTACTACGTCGCTGGCACTGTCGGTCTCATGACGGTACCGGTGATGGGGATTGCTCCGGACTCAAAGGCCTCAGCAGAGAGCGTGTACAATGCCGCACTGGCCCTTGGCATTGCCAACCAGCTCACAAACATCCTCCGAGACGTAGGAGAAGACTCAAGAAGGGGGAGAATATACCTTCCACTGGACGAACTGGCACAGGCGGGTCTGACAGAAGAGGACATATTCAGAGGGAAAGTGACGGATAAATGGAGGAGGTTCATGAAGGGGCAAATCCAGCGCGCCAGGCTCTTCTTTGACGAGGCCGAGAAGGGCGTCATGCATCTAGACTCCGCGAGCAGATGGCCGGTCCTGGCATCGCTGTGGCTGTACAGGCAGATCCTGGACGCCATCGAGGCCAACGACTACAACAACTTCACCAAGCGCGCGTACGTGGGCAAGGCAAAGAAGTTCCTGTCTCTACCGGCCGCGTACGCGAGGGCGGCTCTCTCGCCA TGA

>*TaPSY3_5DL*

ATGCTCTCCACCGGCCGCGCGGTGACCTCGCCCGCGTGCGCTGCCCAGCGCCGGTTCATGGGCGCGGCGCCAAGGACGGTGGTGTTCCTCGCCGGCGACCACGGGAAGCGAGCTGCTGCTTCTGTGGCAGCGCGCGCCGCCGGCCCGTCCCACCCCGGTGTCTGCCTCGAGGCGGCCGCGCCGTGGAGCATGGAGGACGTCGCGGCGCCGGTGACCGTGTCGTACGAGGAGAGCGTGCGCCAGGTCGTGCTCAAGCAGGCGGCGCTCGCGGCCTCGACCACGCCGAGGAAGGCGCGGCTTTCCAGGCGCGACGCCGGCGTGCTGGACGAGGCCTTCGACCGCTGCGGCGCGGTGTGCAAGGAGTACGCCAAGACGTTCTACCTGGCCACGCAGCTCATGACGCCGGAGCGGAGGCGGGCGATCTGGGCAATATACGTGTGGTGCCGTAGGACGGACGAGCTGGTGGACGGCCCGAACGCGGCGCACACGTCGGCGCTGGCGCTGGACCGCTGGGAGTCGCGCCTCGACGGCGTCTTCGCCGGCCGGCCCTACGACATGCTCGACGCGGCGCTGGCCGACGCCGTGGCCGCCTTCCCGGCCGTCGACGAGCGGCCGTTCCGGGACATGGTCCAGGGCATGCGCATGGACCTCGCCAAGTCCCGCTACGCCACCTTCGACGAGCTCTACCTCTACTGCTACCGCGTGGCCGGCACGGTCGGGCTCATGACCGTCCCCGTCATGGGCGTCTCCCCGGGCTCCCAGGCGGGCGTCGAGACGGTGTACGCCGGTGCGCTCGCCCTCGGCGTCGCCAACCAGCTCACCAACATCCTCAGGGACGTCGGCGAGGATGCGAGGAGGGGCAGGATCTACCTGCCGCAGGACGAGCTCGCCACGGCCGGCATCTCCGAGGCGGACATCTTCGCCGGTCGCGTCACCGTCGAGTGGAGGAGCTTCATGAAGGGACAGATTGCGAGGGCCAGGGCCTACTTCCAGCAGGCCGAGCAAGGCGCCGCCGAGCTCAACCAGGAGAGCCGATGGCCGGTGTGGGCATCTCTGCTTCTGTACCGGCAGATCCTGGACGAGATCGAGGCCAACGGCTACGACAACTTCAGCAAGCGAGCCTACGTTCCCAAGGCCAAGAAGCTGGCGGCGCTGCCCAAAGCTTACCTCAGGTCCCTCATGCCTCCTCCTTCTTCGCAGAGGCGCCGTTGA

>*TaPSY3_5AL*

ATGCTCTCCACGGCCCGCGCGGTGACCTCGCCCGCCGCGTGCGCTGCCCAGCACCAGCAGCATGGCGGGAGCCGGTTCGTCGTGTTCCTGGCCGGCGATGACGGGAGGGGAGCTGCCGGTTCCAGCAGCGTGCGTGCGCCGAGGCCGCGCTCTGTGGCAGCGCGTGCCGCCGGCTCGTCCAACCACGCTGTCTGCCTCGAGGCGGCCGCGCCGTGGAGCATGGACTGTGTCGCGGCGCCGGCGACCGTGTCGTACGAGGAGAGCGTGCGCCAGGTCGTGCTCAAGCAGGCGGCGCTCGCGGCCTCGACCACGCCGAGGAAGGCGCGGCACGCCGGCCGTGACGCCGGCGTGCTGGACGCGGCCTTCGACCGCTGCGGCGCGGTGTGCAAGGAGTACGCCAAGACGTTCTACCTGGCCACGCAGCTGATGACGCCGGAACGGAGGCGGGCGATCTGGGCGATATACGTGTGGTGCCGGAGGACGGACGAGCTGGTGGACGGCCCGAACGCGGCGCACACGTCGGCGCTGGCGCTGGACCGCTGGGAGTCGCGCCTCGACGGCGTCTTCGCCGGCCGGCCCTACGACATGCTCGACGCGGCGCTGGCCGACGCCGTGGCCGCCTTCCCGGCGGTCGACGAGCGGCCGTTCCGGGACATGGTCCAGGGGATGCGCATGGACCTCGCCAAGTCCCGCTACGCCACCTTCGACGAGCTCTACCTCTACTGCTACCGCGTGGCCGGCACCGTGGGGCTCATGACCGTCCCCGTCATGGGTGTCTCCCCGGGCTCCCAGGCGGACGTGGAGACGGTGTACGCCGGCGCGCTCGCCCTCGGCGTCGCCAACCAGCTCACCAACATCCTCAGGGACGTCGGCGAGGATGCGAGGAGGGGAAGGATCTACCTGCCGCAGGACGAGCTAGCCATGGCCGGCATCTCCGAGGCGGACATCTTCGCCGGCCGTGTCACCGACGAGTGGAGGAGCTTCATGAAGGGACAGATCGCGAGGGCCAGGGCCTACTTCAAACAAGCCGAGCAAGGCGCCGCCGAGCTCAACCAGGAGAGCCGATGGCCGGTGTGGGCATCTCTGCTTCTGTACCGGCAGATCCTGGACGAGATCGAGGCCAACGGCTACGACAACTTCAGCAAGCGGGCCTATGTTCCCAAGGCCAAGAAGCTGGCGGCGCTGCCCAAAGCTTACCTCAGGTCCCTCATGCCTCCTCCTTCTTCGCAGAGGCGCCGTTGA

>*TaPSY3_5BL*

ATGCTCTCCACCGGCCGCGCGGTGACCTCGCCCGCGCGCGCTGCCCAGCACCGGTTCATGGGCGCACCGAGGACGGTGGTGTTCCTCGCCGGCGACCACGGGAAGCGAGCTGCTGCTTCTGTGGCAGCGCGCGCCGCGGGCTCGTCCCACCCCGCTGTCTGCCTCGAGGCGGCCGCGCCATGGAGCATGGACTGTGTCGCGGCGCCGGCGACCGTGTCGCACGAGGAGAGCGTGCGCCAGGTCGTGCTCAAGCAGGCGGCGCTCGCGGCCTCGACCACGCCGAGGAAGGCGCGGCTCGCCGGGCGCGACGCCGGCGTGCTGGACGCGGCCTTCGCGCGCTGCGGCGCGGTGTGCAAGGAGTACGCCAAGACCTTCTACCTGGCCACGCAGCTCATGACGCCGGAGAGACGGCGGGCGATCTGGGCGATATACGTGTGGTGCCGGAGGACGGACGAGCTGGTGGACGGCCCGAACGCGGCGCACACGTCGGCGCTGGCGCTGGACCGCTGGGAGTCGCGCCTCGACGGCGTCTTCGCCGGCCGGCCCTACGACATGCTGGACGCGGCGCTGGCCGACGCCGTGGCCGCCTTCCCGGCCGTCGACGAGCGGCCGTTCCGGGACATGGTCCAGGGCATGCGCATGGACCTCGCCAAGTCCCGCTACGCCACCTTCGACGAGCTCTACCTCTACTGCTACCGCGTGGCCGGCACCGTCGGGCTCATGACCGTCCCCGTCATGGGCGTCTCCCCGGGCTCCCAGGCGGACGTCGAGACGGTGTACGCCGGCGCGCTCGCCCTCGGCGTCGCCAACCAGCTCACCAACATCCTCAGGGACGTCGGCGAGGATGCGAGGAGGGGCAGGATCTACCTGCCGCAGAACGAGCTGGCCACGGCCGGCATCTCCGAGGCGGACATCTTCGCCGGCCGCGTCACCGACGAGTGGAGGAGCTTCATGAAGGGCCAGATCGCGAGGGCCAGGGCCTACTTCCAGCAGGCCGAGCAAGGCGCCGCCGAGCTCAACCAGGAGAGCCGATGGCCGGTGTGGGCATCTCTGCTTCTGTACCGTCAGATCCTGGACGAGATCGAGGCCAACGGCTACGACAACTTCAGCAAGCGGGCCTATGTTCCCAAGGCCAAGAAGCTGGCGGCGCTGCCCAAAGCTTACCTCAGATCCCTCATGCCCCCTCCTTCGCAGACGCGCCGTTGA
