# Supplementary material for: Characterization and Expression Analysis of Phytoene Synthase from Bread Wheat (Triticum aestivum L.)
Source: PLoS One. 2016 Oct 3;11(10):e0162443. doi: 10.1371/journal.pone.0162443 (PMC5047459; doi:10.1371/journal.pone.0162443)
Supplement: S2 File — (A) Alignment of TaPSY1 located on the long arm of group 7 chromosome. Percent identity between the sequences located on 7A:7B, 7A:7D, and 7B:7D are 97%, 94%, and 90%, respectively. (B) Alignment of TaPSY2 located on the short arm of group 5 chromosome. Percent identity between the sequences located on 5A:5B, 5A:5D, and 5B:5D are 95%, 96%, and 96%, respectively. (C) Alignment of TaPSY3 located on the long arm of group 5 chromosome. Percent identity between the sequences located on 5A:5B, 5A:5D, and 5B:5D are 89%, 92%, and 95%, respectively. (DOC) [file pone.0162443.s002.doc]

**S2 File.** Alignment and similarity analysis of identified *TaPSY* genes encoded by homoeologous sequences from A, B and D subgenomes of *T. aestivum.* (A) Alignment of *TaPSY1* located on the long arm of group 7 chromosome. Percent identity between the sequences located on 7A:7B, 7A:7D, and 7B:7D are 97%, 94%, and 90%, respectively. (B) Alignment of *TaPSY*2 located on the short arm of group 5 chromosome. Percent identity between the sequences located on 5A:5B, 5A:5D, and 5B:5D are 95%, 96%, and 96%, respectively. (C) Alignment of *TaPSY*3 located on the long arm of group 5 chromosome. Percent identity between the sequences located on 5A:5B, 5A:5D, and 5B:5D are 89%, 92%, and 95%, respectively.

**(A)**

**
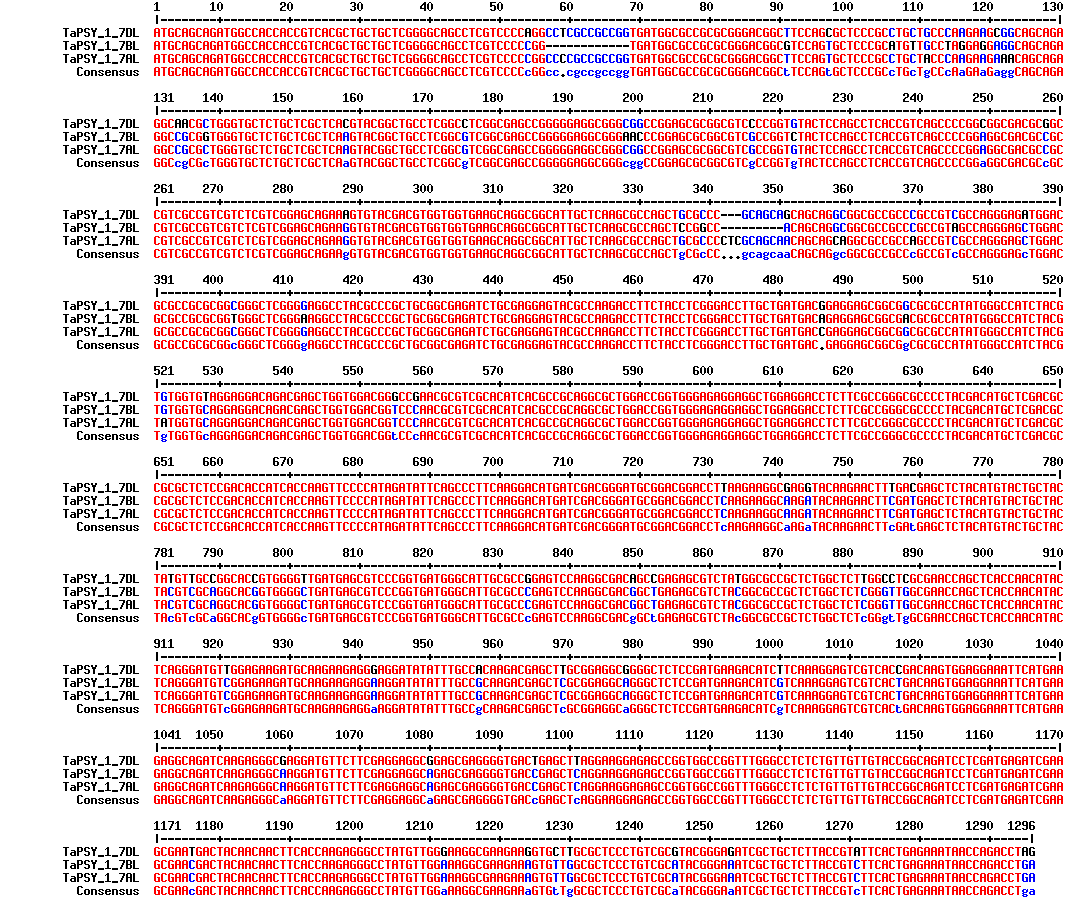
**

**(B)**

**
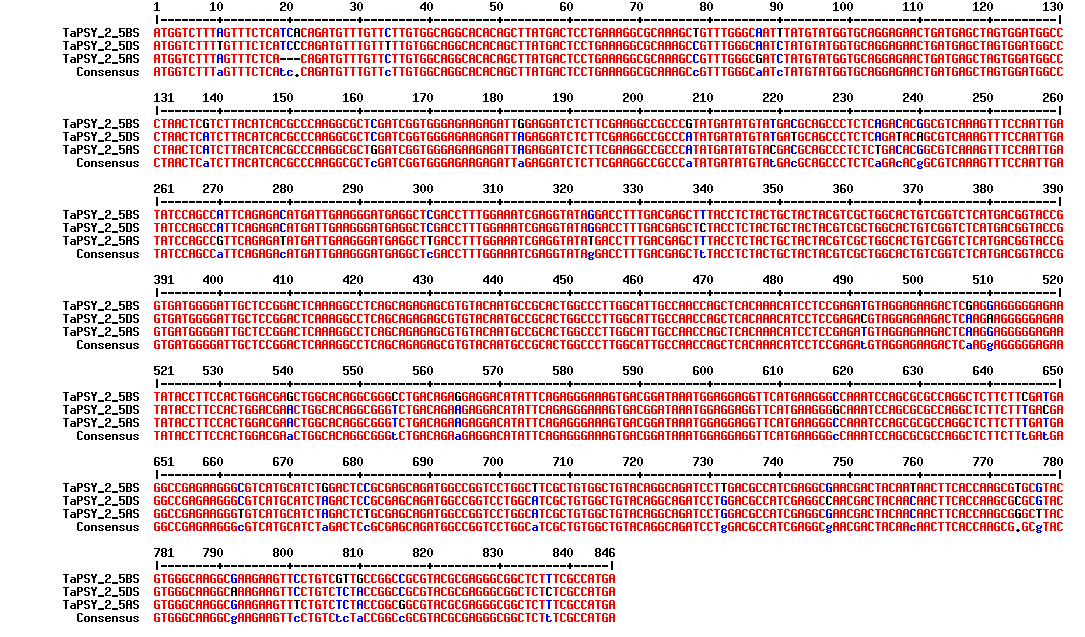
**

**(C)**

**
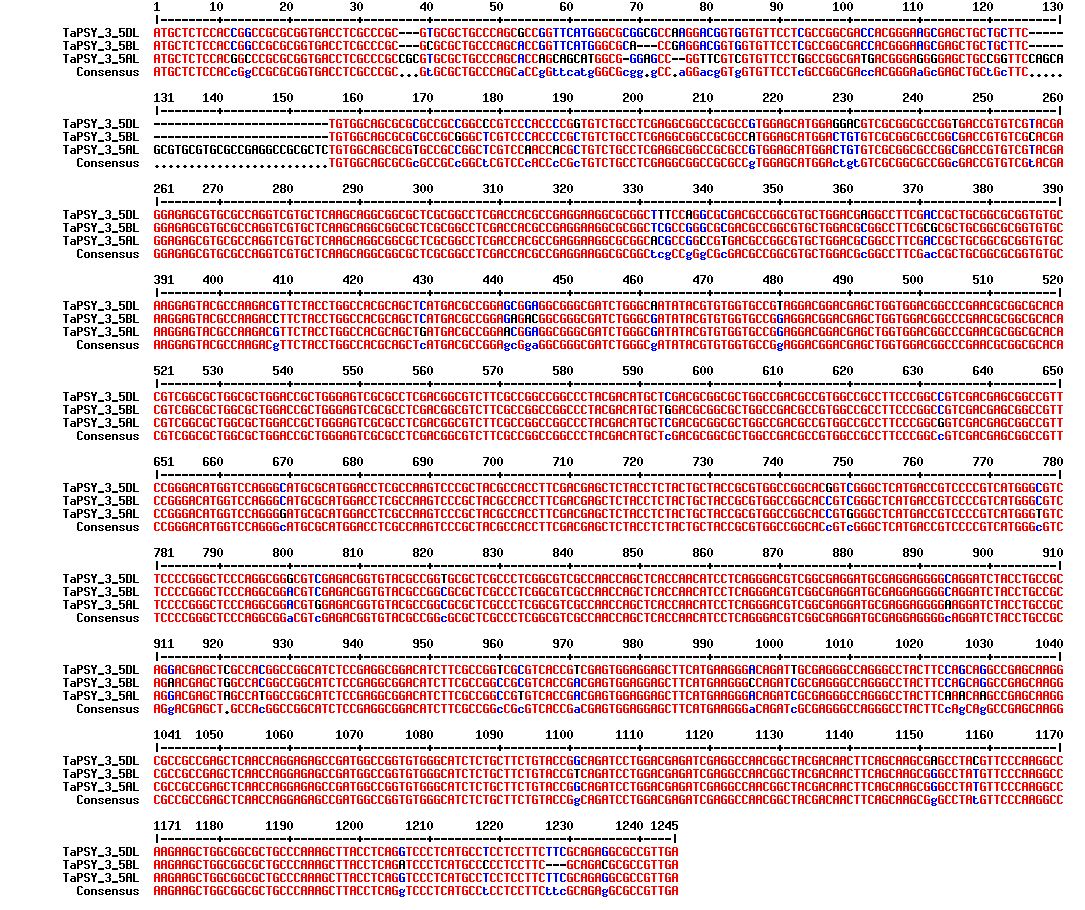
**
