# Supplementary material for: Characterization and Expression Analysis of Phytoene Synthase from Bread Wheat (Triticum aestivum L.)
Source: PLoS One. 2016 Oct 3;11(10):e0162443. doi: 10.1371/journal.pone.0162443 (PMC5047459; doi:10.1371/journal.pone.0162443)
Supplement: S3 File — (A) Alignment of TaPSY1 located on the long arm of group 7 chromosome. Percent identity between 7A:7B, 7A:7D, and 7B:7D are 96%, 96%, and 95%, respectively. (B) Alignment of TaPSY2 located on the short arm of group 5 chromosome. Percent identity between 5A:5B, 5A:5D, and 5B:5D are 98%, 97%, and 99%, respectively. (C) Alignment of TaPSY3 located on the long arm of group 5 chromosome. Percent identity between 5A:5B, 5A:5D, and 5B:5D are 93%, 91%, and 95%, respectively. (DOC) [file pone.0162443.s003.doc]

**S3 File.** Alignment and similarity analysis of amino acid sequences of TaPSY proteins encoded by homoeologous sequences from A, B, and D subgenomes of *T. aestivum.* (A) Alignment of TaPSY1 located on the long arm of group 7 chromosome. Percent identity between 7A:7B, 7A:7D, and 7B:7D are 96%, 96%, and 95%, respectively. (B) Alignment of TaPSY2 located on the short arm of group 5 chromosome. Percent identity between 5A:5B, 5A:5D, and 5B:5D are 98%, 97%, and 99%, respectively. (C) Alignment of TaPSY3 located on the long arm of group 5 chromosome. Percent identity between 5A:5B, 5A:5D, and 5B:5D are 93%, 91%, and 95%, respectively.

**(A)**

**
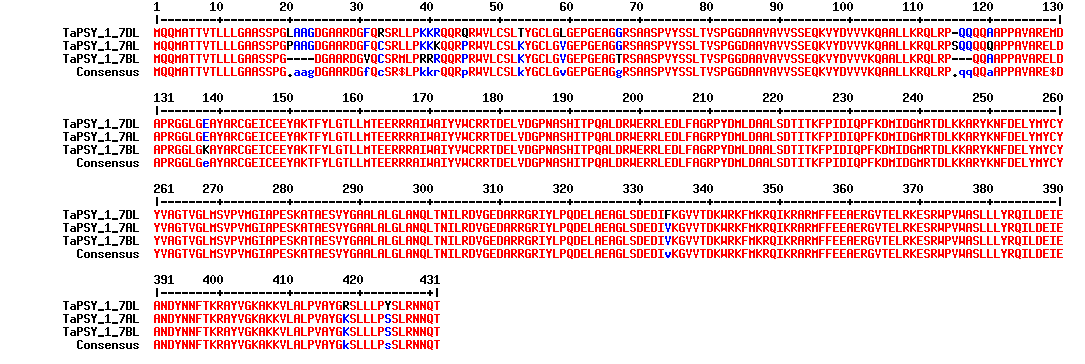
**

**(B)**

**
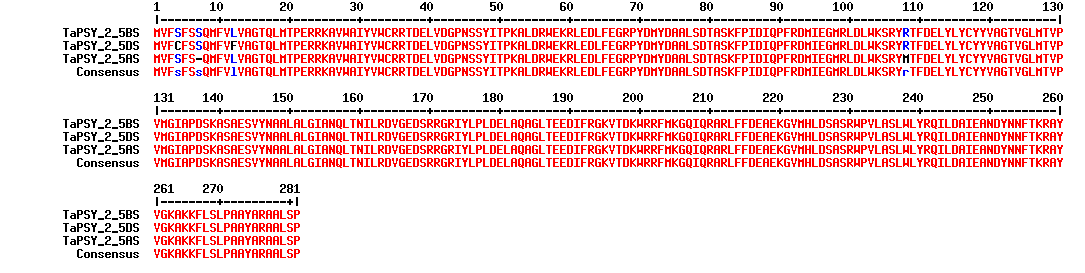
**

**(C)**

**
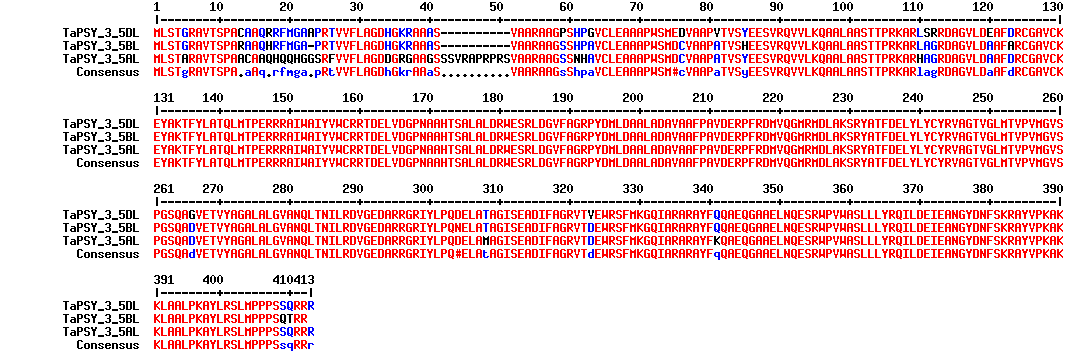
**
