# Supplementary material for: Characterization and Expression Analysis of Phytoene Synthase from Bread Wheat (Triticum aestivum L.)
Source: PLoS One. 2016 Oct 3;11(10):e0162443. doi: 10.1371/journal.pone.0162443 (PMC5047459; doi:10.1371/journal.pone.0162443)
Supplement: S1 Table — (DOCX) [file pone.0162443.s004.docx]

| Gene | Primers name | Primer sequences (5’- 3’) |
| --- | --- | --- |
| *Phytoene synthase1_7AL* | *TaPSY*_1_Fp | ATCGATATG GCCACCACCGTCACGC |
|  | *TaPSY*_1_Rp | ACTAGTCTAGGTCTGGGTTATTTCTCAGTG |
| *Phytoene synthase2_5AS* | *TaPSY*_2_Fp | ATCGATATGGTCTTTAGTTTCTCATCACAGATG |
|  | *TaPSY*_2_Rp | ACTAGTTCATGGCGAAAGAGCCGCCC |
| *Phytoene synthase3_5AL* | *TaPSY*_3_Fp | ATCGATATGCTCTCCACCGGCCGC |
|  | *TaPSY*_3_Rp | ACTAGTTCAACGGCGCCTCTGCG |

**S1 Table.** List of primers used for the amplification of *TaPSY* genes.
