# Supplementary material for: Characterization and Expression Analysis of Phytoene Synthase from Bread Wheat (Triticum aestivum L.)
Source: PLoS One. 2016 Oct 3;11(10):e0162443. doi: 10.1371/journal.pone.0162443 (PMC5047459; doi:10.1371/journal.pone.0162443)
Supplement: S2 Table — (DOCX) [file pone.0162443.s005.docx]

**S2 Table.** List of primers used for used for quantitative real-time PCR analysis of *TaPSY* genes.

| Gene | Primers name | Primer sequences (5’- 3’) |
| --- | --- | --- |
| *Phytoene synthase1_7L* | *TaPSY*_1_Fp | TGCTCGCTCACGTACGGCTGC |
|  | *TaPSY*_1_Rp | GCTCCGACGAGACGACGGCG |
| *Phytoene synthase2_5S* | *TaPSY*_2_Fp | GTGGCAGGCACACAGCTTATGACT |
|  | *TaPSY*_2_Rp | TCCCACCGATCGAGCGCCTTG |
| *Phytoene synthase3_5L* | *TaPSY*_3_Fp | TGGCCGACGCCGTGGCCGC |
|  | *TaPSY*_3_Rp | GAGACGCCCATGACGGGGACGG |
| *ADP ribosylation factor (ARF)* | *TaARF_*Fp | TGATAGGGAACGTGTTGTTGAGGC |
|  | *TaARF_*Rp | AGCCAGTCAAGACCCTCGTACAAC |
