# Supplementary material for: Characterization and Expression Analysis of Phytoene Synthase from Bread Wheat (Triticum aestivum L.)
Source: PLoS One. 2016 Oct 3;11(10):e0162443. doi: 10.1371/journal.pone.0162443 (PMC5047459; doi:10.1371/journal.pone.0162443)
Supplement: S3 Table — (DOCX) [file pone.0162443.s006.docx]

**S3 Table.** Similarity analysis between identified *TaPSY* gene sequences.

|  | ***PSY1_7AL*** | ***PSY1_7BL*** | ***PSY1_7DL*** | ***PSY2_5AS*** | ***PSY2_5BS*** | ***PSY2_5DS*** | ***PSY3_5AL*** | ***PSY3_5BL*** | ***PSY3_5DL*** |
| --- | --- | --- | --- | --- | --- | --- | --- | --- | --- |
| ***PSY1_7AL*** | 100 | 97.09 | 94.50 | 65.83 | 65.95 | 65.72 | 57.97 | 59.03 | 58.08 |
| ***PSY1_7BL*** |  | 100 | 90.19 | 65.95 | 65.83 | 65.48 | 57.81 | 59.03 | 58.41 |
| ***PSY1_7DL*** |  |  | 100 | 67.14 | 66.78 | 67.02 | 57.72 | 59.28 | 58.33 |
| ***PSY2_5AS*** |  |  |  | 100 | 95.61 | 96.20 | 58.60 | 58.36 | 57.88 |
| ***PSY2_5BS*** |  |  |  |  | 100 | 96.09 | 59.10 | 58.98 | 58.74 |
| ***PSY2_5DS*** |  |  |  |  |  | 100 | 58.27 | 58.03 | 57.91 |
| ***PSY3_5AL*** |  |  |  |  |  |  | 100 | 89.46 | 92.40 |
| ***PSY3_5BL*** |  |  |  |  |  |  |  | 100 | 95.60 |
| ***PSY3_5DL*** |  |  |  |  |  |  |  |  | 100 |
