# Supplementary material for: Characterization and Expression Analysis of Phytoene Synthase from Bread Wheat (Triticum aestivum L.)
Source: PLoS One. 2016 Oct 3;11(10):e0162443. doi: 10.1371/journal.pone.0162443 (PMC5047459; doi:10.1371/journal.pone.0162443)
Supplement: S4 Table — (DOCX) [file pone.0162443.s007.docx]

**S4 Table.** Similarity analysis between identified TaPSY protein sequences.

|  | **PSY1_7AL** | **PSY1_7BL** | **PSY1_7DL** | **PSY2_5AS** | **PSY2_5BS** | **PSY2_5DS** | **PSY3_5AL** | **PSY3_5BL** | **PSY3_5DL** |
| --- | --- | --- | --- | --- | --- | --- | --- | --- | --- |
| **PSY1_7AL** | 100 | 96.69 | 96.74 | 75.71 | 75.44 | 75.80 | 56.17 | 57.10 | 56.82 |
| **PSY1_7BL** |  | 100 | 95.28 | 75.71 | 75.44 | 75.80 | 55.93 | 57.10 | 56.07 |
| **PSY1_7DL** |  |  | 100 | 76.42 | 76.15 | 76.51 | 56.90 | 57.85 | 57.56 |
| **PSY2_5AS** |  |  |  | 100 | 98.57 | 97.85 | 66.42 | 66.07 | 66.07 |
| **PSY2_5BS** |  |  |  |  | 100 | 99.28 | 66.19 | 65.83 | 65.83 |
| **PSY2_5DS** |  |  |  |  |  | 100 | 66.54 | 66.19 | 66.19 |
| **PSY3_5AL** |  |  |  |  |  |  | 100 | 93.01 | 91.31 |
| **PSY3_5BL** |  |  |  |  |  |  |  | 100 | 95.01 |
| **PSY3_5DL** |  |  |  |  |  |  |  |  | 100 |
